# Genome assembly catalog for species in the Japanese Red List: unlocking endangered biodiversity through genomic inventory

**DOI:** 10.12688/f1000research.149793.1

**Published:** 2024-06-05

**Authors:** Kirill Kryukov, Naoyuki Nakahama, Shigehiro Kuraku

**Affiliations:** 1 Bioinformation and DDBJ Center, National Institute of Genetics, Mishima, Shizuoka, 411-8540, Japan; 2 Center for Genome Informatics, Joint Support-Center for Data Science Research, Research Organization of Information and Systems, Mishima, Shizuoka, 411-8540, Japan; 3 Division of Ecological Restoration, The Museum of Nature and Human Activities, Sanda, Hyogo, 669-1546, Japan; 4 Institute of Natural and Environmental Sciences, University of Hyogo, Sanda, Hyogo, 669-1546, Japan; 5 Molecular Life History Laboratory, National Institute of Genetics, Mishima, Shizuoka, 411-8540, Japan; 6 Department of Genetics, Sokendai Graduate University for Advanced Studies, Mishima, Shizuoka, 411-8540, Japan

**Keywords:** Biodiversity, Whole Genome Assembly, Red List, Japanese Biota, Japanese Fauna

## Abstract

Improvements in DNA sequencing technology are allowing the dramatic increase of whole genome data for a wide variety of species. Such genome sequence data can assist the monitoring of intraspecific genetic diversity, but is often lacking for threatened species. In this project, we focused on the national Red List, a catalog of extinct and threatened species, issued by the Japanese government. We combined the data included in it with the record of genome assembly in NCBI and tabulated the assembly availability of the species in the list. The combined data shows a low percentage (2.1%) of the availability of whole genome sequence data for the taxa ranked on the Japanese Red List as well as a strong bias towards mammals and birds in Animalia and vascular plants in Plantae. Our data presentation highlights potential systematic limitations in genome sequencing (e.g., budget for sequencing large genomes of amphibians) and instructs future policies including which taxon needs more effort for genome sequencing. The resultant tables are available in the original website https://treethinkers.nig.ac.jp/redlist/ and are regularly updated.

## Introduction

Genome DNA sequences do not only instruct cellular and other phenomena through genetic readout but also contain the valuable information of genetic diversity when they are analyzed on a population level. Investigation of genetic diversity serves as an irreplaceable navigator of conservation biology ( Theissinger et al., 2023), which has been facilitated by recent development of ‘reduced representation sequencing’ methods targeting individuals ( Luikart et al., 2018). However, its feasibility largely relies on the availability of whole genome sequences to be used as ‘reference’ sequences.

Whole genome sequences of endangered species have various advantages in conducting conservation studies on endangered species. First, whole genome information can greatly contribute to the use of genetic information from historical specimens. The use of molecular information from past museum specimens, which is known as “Museomics”, has attracted much attention ( Fong et al. 2023). In recent years, there have been more studies using museum specimens for conservation genomics ( Nakahama 2021). Therein, whole-genome resequencing is a powerful tool. Second, genetic load is quantified from genome information. It is currently difficult to elucidate the mechanism of inbreeding depression itself, as the function and expression mechanism of each deleterious gene is largely unknown. However, it is possible to quantify the genetic load by estimating the amount of deleterious mutations from annotated whole-genome information or transcriptome data ( Hamabata et al. 2019; Dussex et al. 2021; Tian et al. 2022; Yoshida et al., 2020). Genomic information also contributes to recent high-resolution estimation of population demography (reviewed in Nadachowska-Brzyska et al. 2022). Population demography is expected to contribute significantly to the understanding of the natural history of endangered species, as well as to the establishment of conservation units and the determination of conservation policy.

The prevalence of massively parallel sequencing technologies has enabled the acquisition of whole genome sequence information for diverse species. This type of effort has further been accelerated by world-wide trends of biodiversity genomics led by Earth BioGenome Project (EBP) ( Gupta 2022; Lewin et al., 2022), and some projects under the EBP are dedicated to promote this trend in particular districts of the world (e.g., Shaffer et al., 2022). Even though whole genomes have been sequenced for a number of species, some of them remain as contigs that have not undergone further steps to build up longer DNA sequences towards a chromosome scale. Prioritization of our effort in conservation biology should be preceded by the identification of ‘cold spots’ based on the listing of potential species requiring conservation effort and monitoring the current status of whole genome sequencing for those species.

Japan has unique fauna and flora and is selected as a “biodiversity hotspot”, but biodiversity in Japan is experiencing rapid declines ( Marchese 2015; Kobayashi et al. 2019). In 2020, the Ministry of the Environment of Japan (2020) issued an updated version of the Japanese Red List, which included 5,748 taxa, of which 3,716 are categorized as Critically Endangered (CR), Endangered (EN), and Vulnerable (VU). For some of these species, particularly those at high risk of extinction, whole genome sequences have been determined, and conservation genetic studies have accumulated ( Nakahama et al. 2022). To facilitate conservation research on endangered species using genome sequences, it is crucial to monitor the current status and tendency of whole genome sequencing for different taxa to prioritize future policies.

Recognizing the importance of whole genome data and the urgency of the ongoing biodiversity crisis, we decided to start monitoring the availability of assembled genome data for all species in the Japanese Red List. Together with genome data, we also monitor the presence of Red List species in major taxonomic databases. In this paper we describe the structure and methods we use for maintaining our regularly updated resource, which is available online at https://treethinkers.nig.ac.jp/redlist/.

## Methods

### Data sources

We use the Japanese Red List, 2020 edition, published by the Ministry of the Environment, Japan. We use the digital copy of the Red List provided by the IKIMONO LOG ( https://ikilog.biodic.go.jp/). The Red List data is divided in 13 separate csv files, downloadable at https://ikilog.biodic.go.jp/Rdb/booklist.

The NCBI Taxonomy database was obtained from the NCBI server at https://ftp.ncbi.nlm.nih.gov/pub/taxonomy/. For each update, we automatically download the latest dump of the NCBI Taxonomy database. For the GBIF database, we downloaded the latest version of the “GBIF Backbone Taxonomy”, released on August 28, 2023, from the GBIF Datasets page ( https://www.gbif.org/dataset/ => GBIF Backbone Taxonomy => Download). For the iNat taxonomy, we automatically download the latest dataset “iNaturalist Taxonomy DarwinCore Archive” from https://www.inaturalist.org/pages/developers during each update.

For the NCBI genome assembly information, we use the NCBI Datasets command line tool ( https://www.ncbi.nlm.nih.gov/datasets/docs/v2/download-and-install/) to download genome summaries for all taxa related at up to the family level to Red List entries. The command we use is “datasets summary genome taxon ID”. We automatically download the latest summaries during each update.

### Data processing

The original csv files of the Red List used two different text encodings: 10 files used the UTF-8 encoding while the other 3 files used the SHIFT-JIS encoding (redlist2020_kairui.csv, redlist2020_invertebrate.csv, and redlist2020_sorui.csv). We converted the SHIFT-JIS-encoded csv files to UTF-8. We share our set of preprocessed csv files at: https://biokirr.com/Japanese-Red-List-Genomes/Red-List-2020-csv-UTF-8.zip.

We use all unique species or subspecies names from these files. Some entries with the LP (Threatened Local Population) conservation status are listed multiple times, namely for each endangered population. We use only one copy of each of such entries.

When we look up the Red List entries in the three taxonomic databases, we first use the entire scientific name of each entry, and try to locate an identically named entry in the taxonomic database. If we can’t find a corresponding taxonomic record, we use synonyms registered in the database (only with the NCBI Taxonomy database). If we still cannot find the taxonomic record, we use our own list of synonyms. Our list of synonyms is shared at: https://biokirr.com/Japanese-Red-List-Genomes/synonyms.txt.

With the NCBI taxonomy database, we additionally look up the species name and genus name for the entries that can’t be located using the complete scientific name.

### Presentation

Most of our data processing is automated, implemented in a Perl script, freely available at https://biokirr.com/Japanese-Red-List-Genomes/Japanese-Red-List-Genomes-processing-script.zip. This includes downloading and decompressing NCBI genome summaries, downloading and decompressing NCBI and iNat taxonomies, parsing the Red List csv files, and generating all our tables and web pages. We use the uPlot JavaScript library ( https://github.com/leeoniya/uPlot) for the timeline chart. We manually initiate the update process periodically, usually at least once a month, and upload the newly generated files to the web server. Our script is available at https://biokirr.com/Japanese-Red-List-Genomes/Japanese-Red-List-Genomes-processing-script.zip. The script is shared under the zlib/libpng license and is free to use, modify and distribute.

## Results

All our data was produced by cross-referencing the Japanese Red List (latest edition 2020 as of March 2024) with the major taxonomic and genomic databases. We obtained taxonomic and genomic data from NCBI (National Center for Biotechnology Information, https://www.ncbi.nlm.nih.gov/), and additional taxonomic data from GBIF (Global Biodiversity Information Facility, https://www.gbif.org/) and iNat (iNaturalist, https://www.inaturalist.org/). Our results are available online without restrictions at the following url: https://treethinkers.nig.ac.jp/redlist/. The main page includes summary statistics, and links to more detailed tables. We update the website periodically, to reflect the latest taxonomy and genome information. The date of the latest update is shown at the top of the main page.

**Figure 1.**
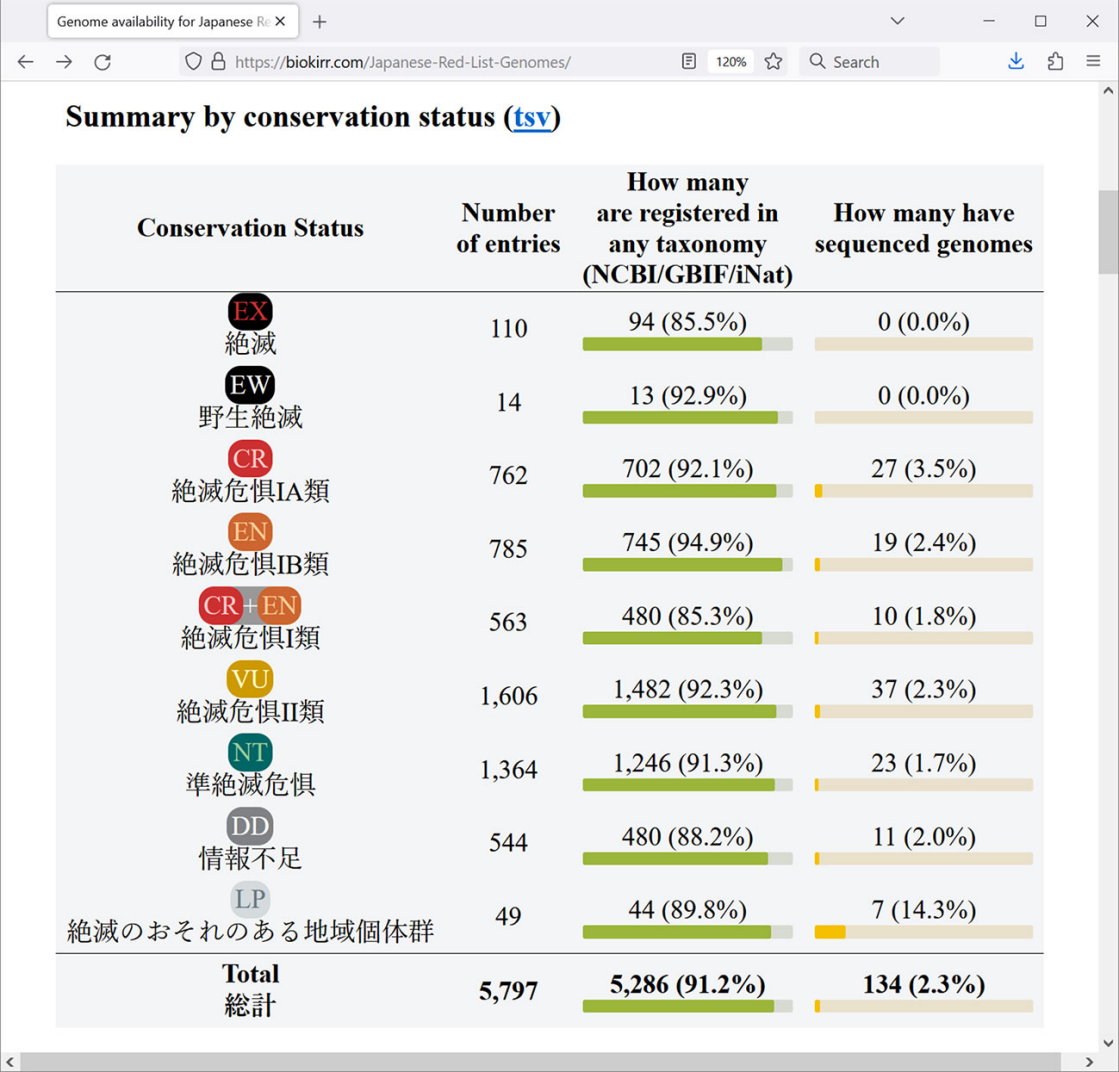
Screenshot of a browser window showing the Red List summary by conservation status. Data is shown as of May 2024.

**Figure 2.**
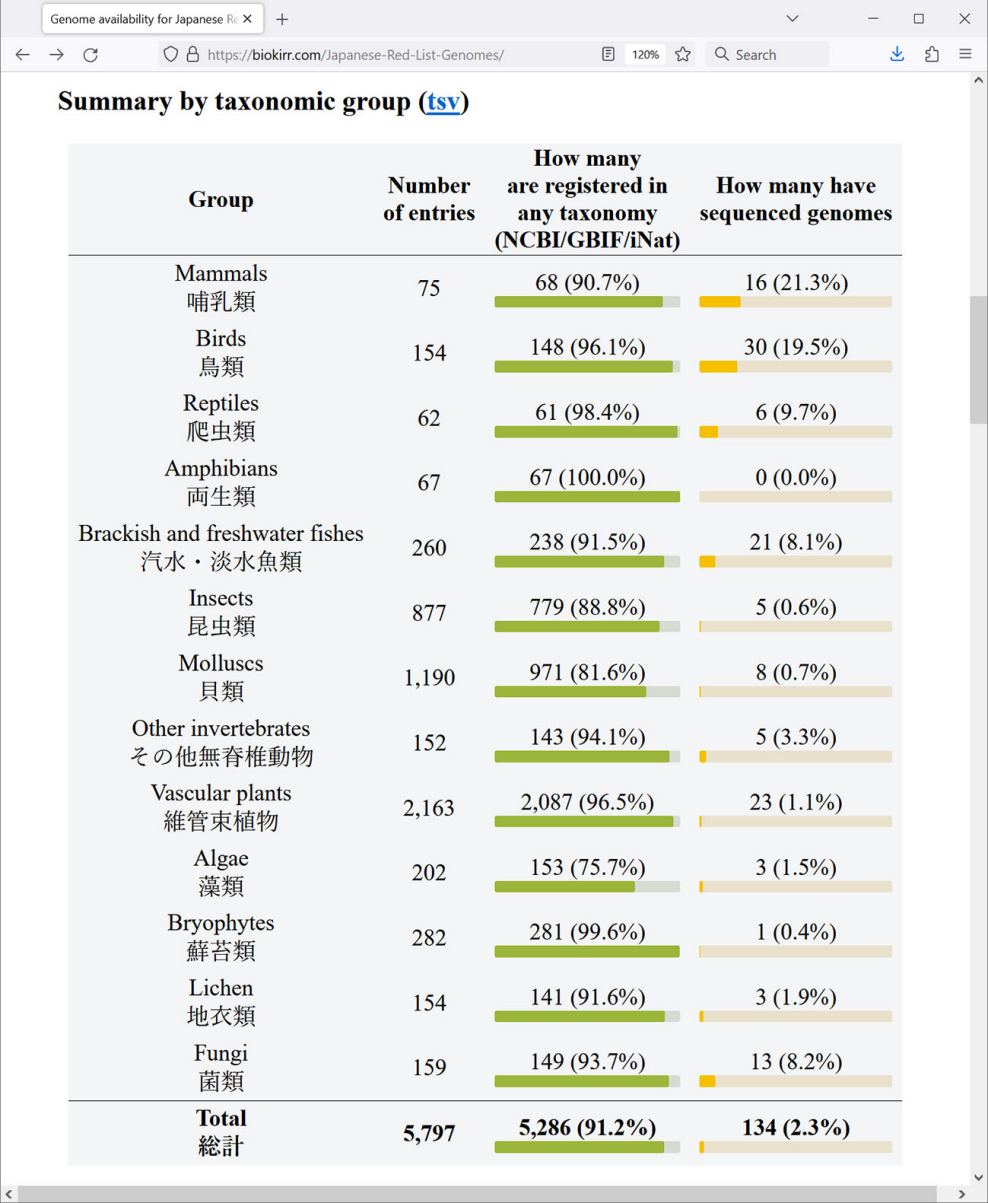
Screenshot of a browser window showing the Red List summary by taxonomic group. Data is shown as of May 2024.

Registering whole genome sequences in public database should be preceded by specifying the organism from which the sequences were derived, which is a typical format of the database. The two main data types presented in our newly established resource are the availability of the Red List entries in taxonomic databases, and the availability of whole genome sequences of those Red List entries. The first table at the main page, “Summary by conservation status” ( Figure 1), shows the total numbers for each conservation status. The next table, “Summary by taxonomic group” ( Figure 2), shows the numbers for the 13 taxonomic groups defined in the Red List. These tables are convenient for seeing how many Red List entries are registered in at least one of the taxonomic databases (either NCBI, GBIF, or iNat), and how many Red List entries already have assembled genomes.

In the following table, “Comparison of taxonomies”, the three taxonomic datasets that we use are compared side by side with each other, in terms of covering the 13 sections of the Red List. The rightmost column again displays the total number of entries that are registered in any of the three taxonomic databases. The next table, “Taxonomy coverage”, shows how many Red List entries have corresponding NCBI Taxonomy nodes of three different ranks: complete name (can be species, or subspecies or a variety), species, and genus.

**Figure 3.**
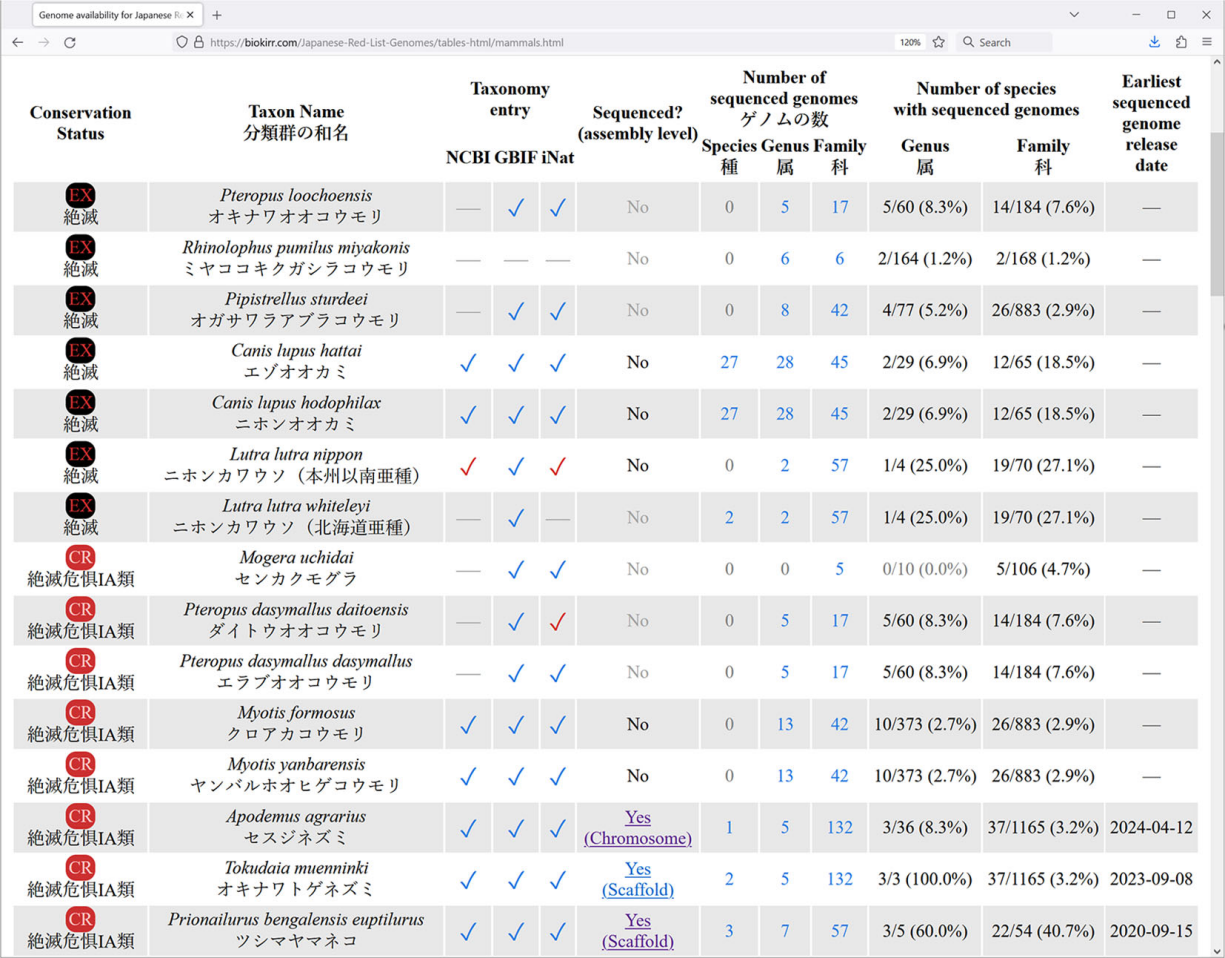
Screenshot of a browser window showing the beginning of the detailed table for the "Mammals" section of the Red List. Data is shown as of May 2024.

Row names (in the first column) in all these summary tables link to detail tables, where taxonomic and genomic information is shown for each individual Red List entry ( Figure 3). All detail tables contain one Red List entry per row, and are structured in the same way. First, the conservation status and taxon names (scientific and Japanese names) are shown. The names are shown exactly as provided in the Red List, including names that are misformatted or contain typos, to make it easier to relate our data with the Red List. The next three columns show whether the entry is registered in the taxonomic databases (NCBI, GBIF, and iNat). If a Red List entry is found in the taxonomic database, its table cell contains a check mark, linked to the corresponding entry in the taxonomic database. The check mark color indicates the method of locating the name in taxonomy. Blue check mark means that the exact scientific name used in the Red List is registered in the taxonomic database. Brown or red check mark means that the Red List entry is registered in the taxonomic database under a different name, and that a synonym was used to find the connection. In such cases the check mark color indicates the source of the synonym: brown check mark means that the synonym is registered in the taxonomic database, red check mark means that the synonym is from our own manually constructed dataset of synonyms.

The “Sequenced? (assembly level)” column shows whether an entry has sequence genome, and the level of the best available assembly (complete > chromosome > scaffold > contig). It also links to the genome assembly pages on the NCBI website. The three columns under the “Number of sequenced genomes” title show the total number of available genomes for organisms in the same species, genus, and family, with the corresponding Red List entry. The next two columns under the “Number of species with sequenced genomes” title show how many distinct species already have sequenced genomes in the same genus and family with the Red List entry. Finally, the last column shows the release date of the earliest available genome assembly for each entry.

**Figure 4.**
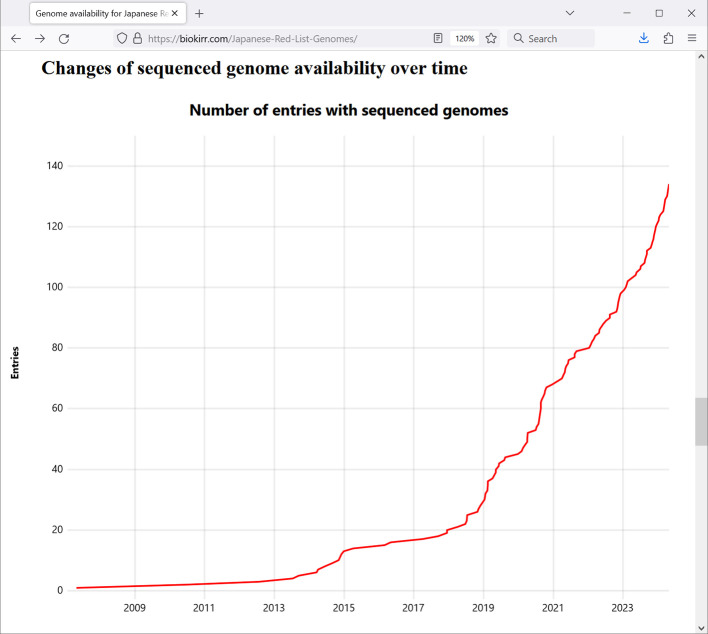
Screenshot of a browser window with the chart showing the change of the number of Red List entries with sequenced genomes by time. Data is shown as of May 2024.

The chart “Changes of sequenced genome availability over time” ( Figure 4) on the main page shows how many Red List entries already had genome sequence data at each point of time. It can be seen that genome sequencing accelerated around the beginning of the year 2019, and is currently continuing with the speed of about 40 newly sequenced entries per year.

The next section on the main page shows the 20 most recently released genomes. Finally, the link “Submitters of sequenced genomes” brings us to the list of organizations that performed genome sequencing, ranked by the number of Red List entries covered. As of May 2024, the Japanese National Institute for Environmental Studies leads the list, with 15 sequenced species (divided into multiple lines because of spelling differences in the registered organization name).

## Discussion

We cross-referenced the Red List with the three major international taxonomic databases: NCBI, GBIF and iNat. NCBI Taxonomy is used for annotating sequence data, and links to all available sequence data in other NCBI databases. GBIF provides literature references and occurrence locations. iNat provides occurrence locations and photographs. These databases are curated independently from each other, and provide different unique data. Together, these three taxonomic resources provide a comprehensive coverage of the worldwide biological diversity. We found that 91.2% of the Red List entries are registered in at least one taxonomic database. It is crucial that 8.9% of Red List entries are not registered in any of the three taxonomic databases that we used. Taxonomic classification is the backbone of biological research, as it enables systematic ways of discussing and describing the relationships between various organisms. Thus, it is expected that all endangered organisms of the Japanese Red List are registered in all major international taxonomic databases.

The web site generated in this project offers a gateway to monitor the availability of whole genome sequence information for (sub) species in the Japanese Red List. As of May 2024, 2.3% of all the entries (species or subspecies) have whole genome assemblies in NCBI. Mammals and birds are relatively better covered groups, with 21.3% and 19.5% of the species’ genomes already sequenced, respectively. Reptiles and fishes have 9.7% and 8.1% of the entries sequenced. Also, fungi have 8.2% of entries sequenced. The rest of the groups are almost completely uncovered by genome data. For example, the taxon Amphibia in our list contains quite a few salamanders endemic to Japan, and their genomes are exceptionally large, exceeding 10 Gb. The technical difficulty is reflected in the unavailability of genome assemblies in this taxon. In total, only 134 Red List entries have assembled genomes, or about 2.3% of all entries. We expect that the rate of genome sequencing will largely increase, and that eventually assembled genomes will cover the entire Red List.

Whole genome data is increasingly important in biological and conservation research, as it can provide a better understanding of endangered organisms, and help in the conservation efforts. Our comprehensive catalog of genomic and taxonomic information for the Japanese Red List will not only be useful for locating genome assemblies, but, importantly, it will also help focusing the future research efforts and efficiently allocating the scarce resources available for genome sequencing projects. We will continue monitoring the available data and updating our website, and similar efforts are anticipated in other regions of the world, in order to fuel preemptive, evidence-based biodiversity conservation.

## Ethics and consent

No personal or otherwise human-related data was used in this study. All data we used is already open and public. Therefore, no ethics-related or consent-related issues are applicable to this study.

## Data Availability

All our data is placed in the public domain, and shared on the website https://treethinkers.nig.ac.jp/redlist/.
