# Genome assembly catalog for species in the Japanese Red List: unlocking endangered biodiversity through genomic inventory

**DOI:** 10.12688/f1000research.149793.2

**Published:** 2024-07-22

**Authors:** Kirill Kryukov, Naoyuki Nakahama, Shigehiro Kuraku

**Affiliations:** 1 Center for Genome Informatics, Joint Support-Center for Data Science Research, Research Organization of Information and Systems, Mishima, Shizuoka, 411-8540, Japan; 2 Bioinformation and DDBJ Center, National Institute of Genetics, Mishima, Shizuoka, 411-8540, Japan; 3 Institute of Natural and Environmental Sciences, University of Hyogo, Sanda, Hyogo, 669-1546, Japan; 4 Division of Ecological Restoration, Museum of Nature and Human Activities, Hyogo, Sanda, Hyogo, 669-1546, Japan; 5 Molecular Life History Laboratory, National Institute of Genetics, Mishima, Shizuoka, 411-8540, Japan; 6 Department of Genetics, Sokendai Graduate University for Advanced Studies, Mishima, Shizuoka, 411-8540, Japan

**Keywords:** Biodiversity, Whole Genome Assembly, Red List, Japanese Biota, Japanese Fauna

## Abstract

Improvements in DNA sequencing technology are allowing the dramatic increase of whole genome data for a wide variety of species. Such genome sequence data can assist the monitoring of intraspecific genetic diversity, but is often lacking for threatened species. In this project, we focused on the national Red List, a catalog of extinct and threatened species, issued by the Japanese government. We combined the data included in it with the record of genome assembly in NCBI and tabulated the assembly availability of the species in the list. The combined data shows a low percentage (2.1%) of the availability of whole genome sequence data for the taxa ranked on the Japanese Red List as well as a strong bias towards mammals and birds in Animalia and vascular plants in Plantae. Our data presentation highlights potential systematic limitations in genome sequencing (e.g., budget for sequencing large genomes of amphibians) and instructs future policies including which taxon needs more effort for genome sequencing. The resultant tables are available in the original website https://treethinkers.nig.ac.jp/redlist/ and are regularly updated.

## Introduction

Genome DNA sequences do not only instruct cellular and other phenomena through genetic readout but also contain valuable information about genetic diversity when they are analyzed on a population level. Investigation of genetic diversity serves as an irreplaceable navigator of conservation biology ( Theissinger et al., 2023), which has been facilitated by recent development of ‘reduced representation sequencing’ methods targeting individuals ( Luikart et al., 2018). However, its feasibility largely relies on the availability of whole genome sequences to be used as ‘reference’ sequences.

Whole genome sequences of endangered species have various advantages in conducting conservation studies on endangered species. First, whole genome information can greatly contribute to the use of genetic information from historical specimens. The use of molecular information from past museum specimens, which is known as “Museomics”, has attracted much attention ( Fong et al., 2023). In recent years, there have been more studies using museum specimens for conservation genomics ( Nakahama, 2021). Therein, whole-genome resequencing is a powerful tool. Second, genetic load is quantified from genome information. It is currently difficult to elucidate the mechanism of inbreeding depression itself, as the function and expression mechanism of each deleterious gene is largely unknown. However, it is possible to quantify the genetic load by estimating the amount of deleterious mutations from annotated whole-genome information or transcriptome data ( Hamabata et al., 2019; Dussex et al., 2021; Tian et al., 2022; Yoshida et al., 2020). Genomic information also contributes to recent high-resolution estimation of population demography (reviewed in Nadachowska-Brzyska et al., 2022). Population demography is expected to contribute significantly to the understanding of the natural history of endangered species, as well as to the establishment of conservation units and the determination of conservation policy.

The prevalence of massively parallel sequencing technologies has enabled the acquisition of whole genome sequence information for diverse species. This type of effort has further been accelerated by world-wide trends of biodiversity genomics led by Earth BioGenome Project (EBP) ( Gupta, 2022; Lewin et al., 2022), and some projects under the EBP are dedicated to promoting this trend in particular districts of the world (e.g., Shaffer et al., 2022). Even though whole genomes have been sequenced for a number of species, some of them remain as contigs that have not undergone further steps to build up longer DNA sequences towards a chromosome scale. Prioritization of our effort in conservation biology should be preceded by the identification of ‘cold spots’ based on the listing of potential species requiring conservation effort and monitoring the current status of whole genome sequencing for those species.

Japan has unique fauna and flora and is selected as a “biodiversity hotspot”, but biodiversity in Japan is experiencing rapid declines ( Marchese, 2015; Kobayashi et al., 2019). In 2020, the Ministry of the Environment of Japan (2020) reassessed the risk of extinction for approximately 58,000 taxa of Japanese wildlife and issued an updated version of the Japanese Red List, which included 5,748 taxa, of which 3,716 are categorized as Critically Endangered (CR), Endangered (EN), and Vulnerable (VU). In addition, the Ministry of the Environment Marine Life Red List (2017) assessed the risk of extinction for approximately 10,120 taxa of Japanese marine wildlife. As a result, 443 taxa were listed, of which 56 taxa were assessed as endangered. For some of these species, particularly those at high risk of extinction, whole genome sequences have been determined, and conservation genetic studies have accumulated ( Nakahama et al., 2022). Introducing Red lists has aroused controversies about its biased species selection towards organisms with relatively large sizes or those with frequent human experience ( Possingham et al., 2002; Cazalis et al., 2022; Régnier et al., 2009; Goodsell et al., 2024), but they serve as one official proxy out of limited existing resources. To facilitate conservation research on endangered species using genome sequences, it is crucial to monitor the current status and tendency of whole genome sequencing for different taxa to prioritize future policies.

Recognizing the importance of whole genome data and the urgency of the ongoing biodiversity crisis, we decided to start monitoring the availability of assembled genome data for all species in the Japanese Red List as a proxy for a full listing of endangered species. We also monitor the presence of Red List species in major taxonomic databases. In this paper we describe the structure and methods we use for maintaining our regularly updated resource, which is available online at https://treethinkers.nig.ac.jp/redlist/.

## Methods

### Data sources

We use the Japanese Terrestrial Red List, 2020 edition, and the Japanese Marine Red list, 2017 edition, published by the Ministry of the Environment, Japan. We use the digital copy of the Red List available at https://ikilog.biodic.go.jp/Rdb/booklist.

For each update, we download the NCBI Taxonomy database from https://ftp.ncbi.nlm.nih.gov/pub/taxonomy/ and the iNat taxonomy from https://www.inaturalist.org/pages/developers. For the GBIF taxonomy, we use the latest available version of the “GBIF Backbone Taxonomy”, released on August 28, 2023, from https://www.gbif.org/dataset/. For COL taxonomy we use the latest monthly snapshot 2024-06-27 from https://download.checklistbank.org/col/monthly/. We use the 2022-2 version of the IUCN Red List from GBIF ( https://www.gbif.org/dataset/).

For the NCBI genome assembly information, we use the NCBI “datasets” command line tool ( https://www.ncbi.nlm.nih.gov/datasets/docs/v2/download-and-install/) to download genome summaries for all families related to Red List entries. We automatically download the latest summaries during each update.

### Data processing

We first converted the SHIFT-JIS-encoded red list csv files to UTF-8, which we share at: https://biokirr.com/Japanese-Red-List-Genomes/Red-List-2020-csv-UTF-8.zip.

We use all unique organism names from these files. Some entries with the LP (Threatened Local Population) conservation status are listed multiple times, namely for each endangered population. We use only one copy of each of such entries.

When we look up the Red List entries in the four taxonomic databases and in the IUCN Red List, we first use the entire scientific name of each entry, and try to locate an identically named entry in the taxonomic database. If such entry cannot be found, we use synonyms registered in the database. If the corresponding entry still cannot be located, we use our own list of synonyms. Our synonyms are shared at: https://biokirr.com/Japanese-Red-List-Genomes/synonyms.txt.

With the NCBI taxonomy database, we additionally look up the species name and genus name for the entries that cannot be located using the complete scientific name.

### Presentation

Most of our data processing is automated, including downloading genome summaries, downloading and decompressing NCBI and iNat taxonomies, reading the Red List csv files and taxonomy databases, and generating all tables and web pages. We use uPlot ( https://github.com/leeoniya/uPlot) for the timeline chart. We perform the update periodically, usually at least once a month. Our scripts are available at https://biokirr.com/Japanese-Red-List-Genomes/Japanese-Red-List-Genomes-processing-scripts.zip. The scripts are shared under the zlib/libpng license and are free to use, modify and distribute.

## Results

All our data was produced by cross-referencing the Japanese Red Lists with the major taxonomic and genomic databases, and with the IUCN Red List. Our results are available online without restrictions at the following url: https://treethinkers.nig.ac.jp/redlist/. The main page includes summary statistics, and links to more detailed tables. We update the website periodically, to reflect the latest taxonomy and genome information. The date of the latest update is shown at the top of the main page.

**Figure 1.**
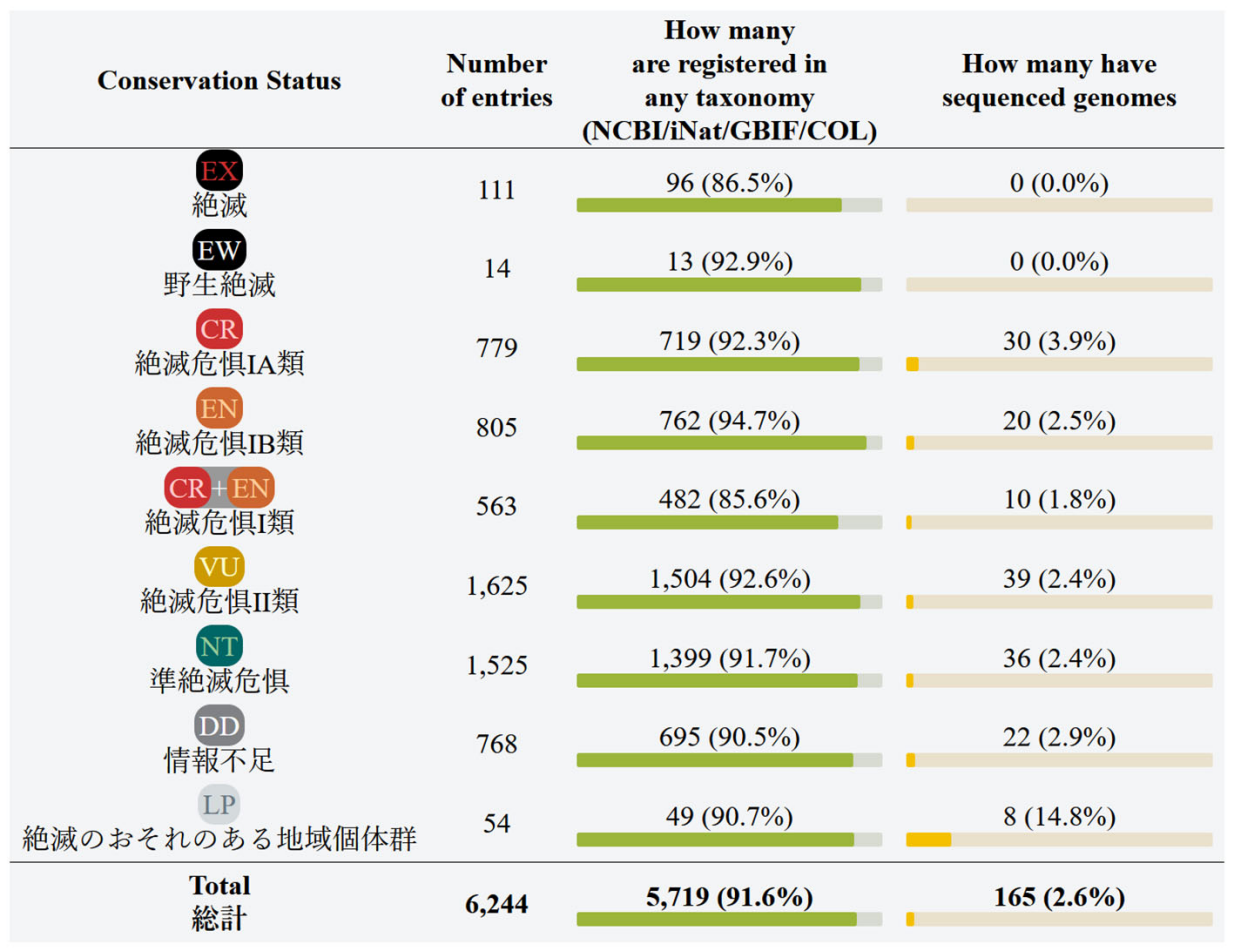
Japanese Red List summary by conservation status (screenshot of the webpage). Data is shown as of July 2024. Conservation statuses: EX = extinct, EW = extinct in the wild, CR = critically endangered, EN = endangered, VU = vulnerable, NT = nearly threatened, DD = data deficient, LP = local population declining. Database names: NCBI = National Center for Biotechnology Information, iNat = iNaturalist, GBIF = Global Biodiversity Information Facility, COL = Catalogue of Life.

**Figure 2.**
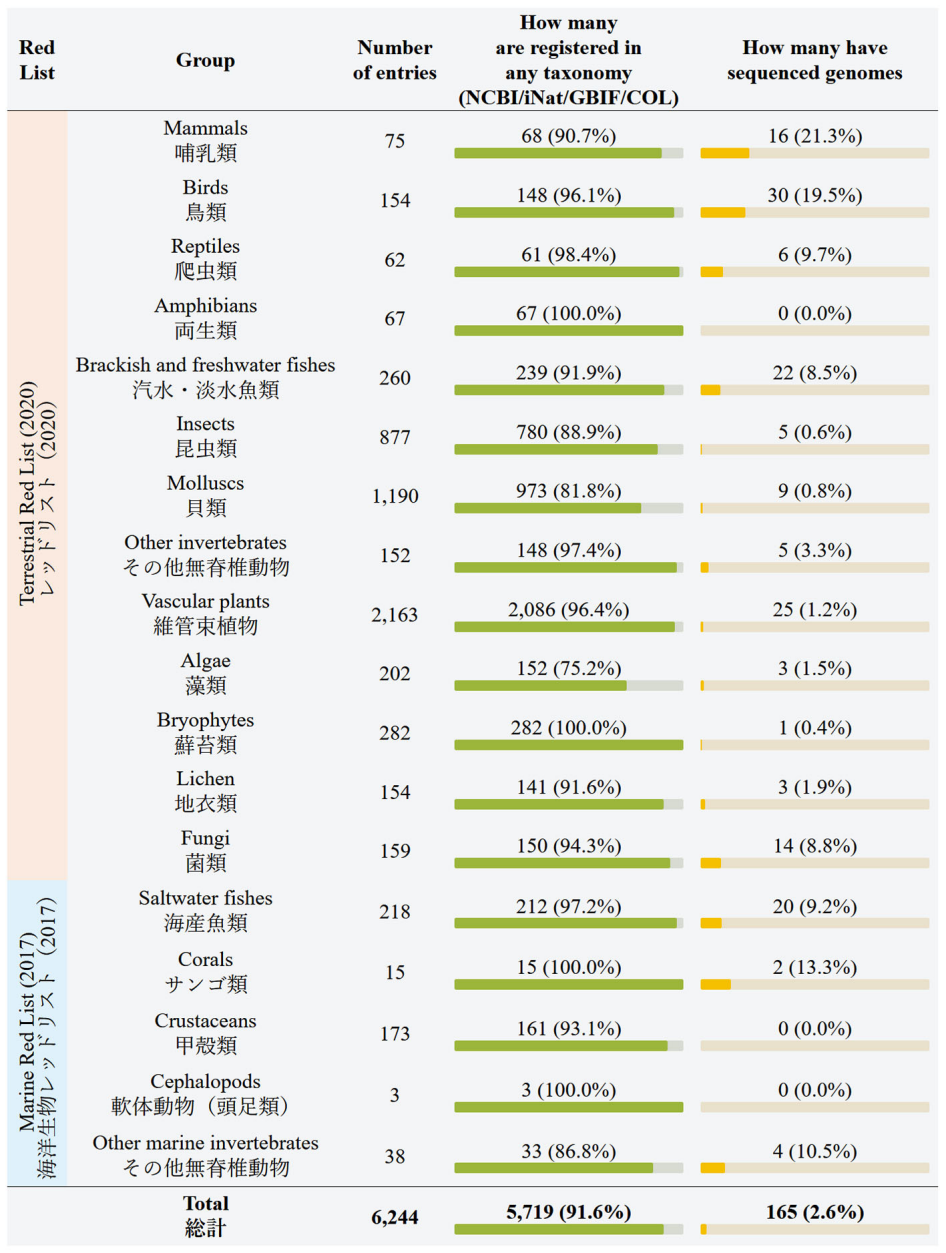
Japanese Red List summary by red list section (screenshot of the webpage). Data is shown as of July 2024. Database names: NCBI = National Center for Biotechnology Information, iNat = iNaturalist, GBIF = Global Biodiversity Information Facility, COL = Catalogue of Life.

Registering whole genome sequences in public database should be preceded by specifying the organism from which the sequences were derived. The two main data types presented in our newly established resource are the availability of the Red List entries in taxonomic databases, and the availability of whole genome sequences of those Red List entries. The first table at the main page, “Summary by conservation status” ( Figure 1), shows the total numbers for each conservation status. The next table, “Summary by red list section” ( Figure 2), shows the numbers for the 18 groups defined in the Red List. These tables are convenient for seeing how many Red List entries are registered in taxonomic databases (NCBI, iNat, GBIF, and COL), and how many Red List entries already have assembled genomes.

**Figure 3.**
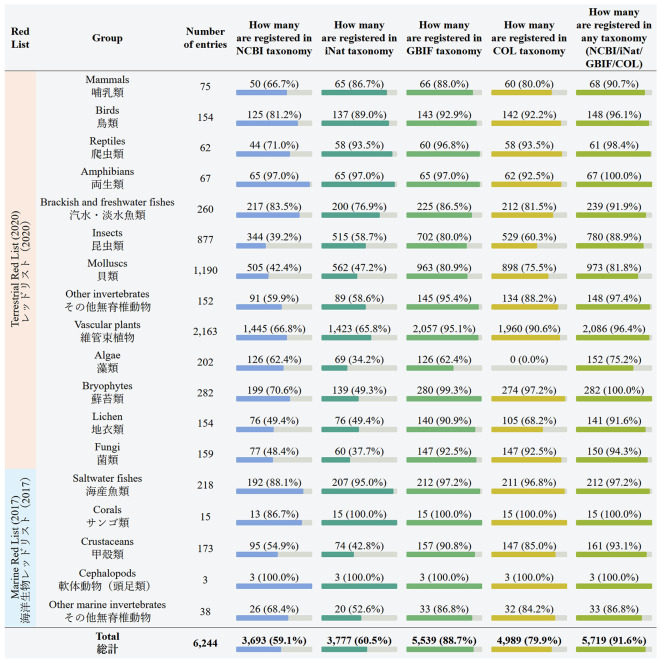
Coverage of the Japanese Red List sections by taxonomy databases (screenshot of the webpage). Data is shown as of July 2024. Database names: NCBI = National Center for Biotechnology Information, iNat = iNaturalist, GBIF = Global Biodiversity Information Facility, COL = Catalogue of Life.

In the following table, “Comparison of taxonomies” ( Figure 3), the four taxonomic datasets that we use are compared side by side with each other, in terms of covering the 18 sections of the Red List. The rightmost column again displays the total number of entries that are registered in any of the four taxonomic databases. The next table, “Comparison with the IUCN Red List”, shows how many of the Japanese Red List entries are registered in the IUCN Red List, and what conservation status they have there.

**Figure 4.**
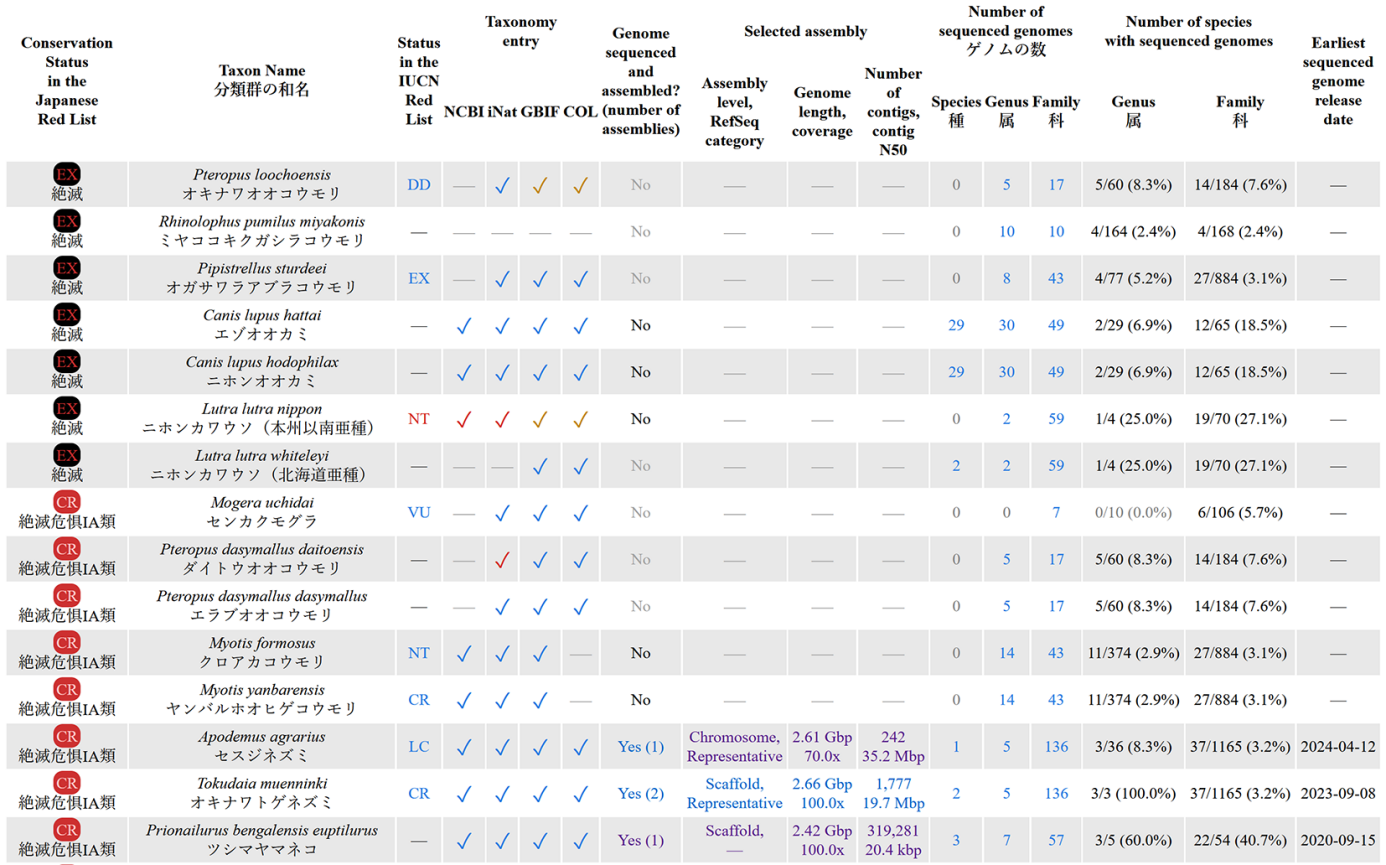
The beginning of the detailed table for the “Mammals” section of the Japanese Red List (screenshot of the webpage). Data is shown as of July 2024. Conservation statuses: EX = extinct, CR = critically endangered, VU = vulnerable, NT = nearly threatened, DD = data deficient, LC = least concern. Database names: IUCN = International Union for Conservation of Nature, NCBI = National Center for Biotechnology Information, iNat = iNaturalist, GBIF = Global Biodiversity Information Facility, COL = Catalogue of Life.

Row names (in the first column) in all these summary tables link to detail tables, where taxonomic and genomic information is shown for each individual Red List entry ( Figure 4). All detail tables display one Red List entry per row, and are structured in the same way. First, the conservation status and taxon names (scientific and Japanese names) are shown. The names are shown exactly as provided in the Japanese Red List. The next column shows if the organism is registered in the IUCN Red List, and its conservation status in that list. The next four columns show whether the entry is registered in the taxonomic databases (NCBI, iNat, GBIF, and COL). If a Red List entry is found in the taxonomic database, its table cell contains a check mark, linked to the corresponding entry in the taxonomic database. The check mark color indicates the method of locating the name in taxonomy. Blue check mark means that the exact scientific name used in the Red List is registered in the taxonomic database. Brown or red check mark means that the Red List entry is registered in the taxonomic database under a different name, and that a synonym was used to find the connection. In such cases the check mark color indicates the source of the synonym: brown check mark means that the synonym is registered in the taxonomic database, red check mark means that the synonym is from our own manually constructed dataset of synonyms.

The next column shows whether an entry has an assembled genome, and the total number of assemblies available. The next three columns show information about the selected best genome assembly of this organism. The data shown include the assembly level (Contig/Scaffold/Chromosome/Complete), RefSeq category of the assembly (Representative/Reference), genome size, sequencing coverage (depth), number of contigs and contig N50 statistics. It also links to the genome assembly pages on the NCBI website. The three columns under the “Number of sequenced genomes” title show the total number of available genomes for organisms in the same species, genus, and family, with the corresponding Red List entry. The next two columns under the “Number of species with sequenced genomes” title show how many distinct species already have sequenced genomes in the same genus and family with the Red List entry. Finally, the last column shows the release date of the earliest available genome assembly for each entry.

**Figure 5.**
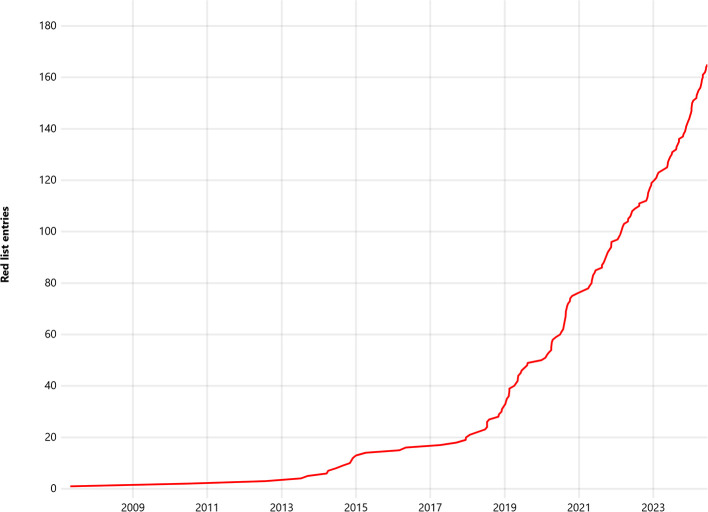
Chart showing the change of the number of Japanese Red List entries with sequenced genomes by time (screenshot of the webpage). Data is shown as of July 2024.

The chart “Changes of red list coverage with genome data over time” ( Figure 5) on the main page shows how many Red List entries already had genome sequence data at each point of time. It can be seen that genome sequencing accelerated around the beginning of the year 2019, and is currently continuing with the speed of about 40 newly sequenced entries per year.

The next section on the main page shows the 20 most recently released genomes. Finally, the link “Submitters of sequenced genomes” brings us to the list of organizations that performed genome sequencing, ranked by the number of Red List entries covered. As of May 2024, the Japanese National Institute for Environmental Studies leads the list, with 15 sequenced entries (divided into multiple lines because of spelling differences in the registered organization name).

## Discussion

We cross-referenced the Red List with the four major international taxonomic databases: NCBI, iNat, GBIF and COL. NCBI Taxonomy is used for annotating sequence data, and links to all available sequence data in other NCBI databases. iNat provides occurrence locations and photographs. GBIF provides literature references and occurrence locations. COL aims to be “The most complete authoritative list of the world’s species”. These databases are curated independently from each other, and provide different unique data. Together, these four taxonomic resources provide a comprehensive coverage of the worldwide biological diversity. We found that 91.6% of the Red List entries are registered in at least one taxonomic database. The remaining 8.4% of Red List entries are not registered in any of the four taxonomic databases that we used. Taxonomic classification is the backbone of biological research, as it enables systematic ways of discussing and describing the relationships between various organisms. Thus, it is expected that all endangered organisms of the Japanese Red List are registered in all major international taxonomic databases.

The web site generated in this project offers a gateway to monitor the availability of whole genome sequence information for (sub) species in the Japanese Red List. As of July 2024, 2.6% of all entries (species or subspecies) have whole genome assemblies in NCBI. Mammals and birds are relatively better covered groups, with 21.3% and 19.5% of the species’ genomes already sequenced, respectively. Reptiles have 9.7% of the entries sequenced. Freshwater and saltwater fishes have 8.5% and 9.2% of the entries sequenced. Also, fungi have 8.8% of entries sequenced. The rest of the groups are almost completely uncovered by genome data. For example, 69% (46/67) of the Amphibia section of the Japanese Red List are salamanders, and their genomes are exceptionally large, exceeding 10 Gbp. The technical difficulty is reflected in the lack of available genome assemblies for this taxon. In total, only 165 Red List entries have assembled genomes, or about 2.6% of all entries. We expect that the rate of genome sequencing will largely increase, and that eventually assembled genomes will cover the entire Red List.

We note that we only monitor NCBI Datasets for genome data at the moment, as it is the largest repository of genome data. Other platforms for sharing genome data exists, such as Ensembl ( https://ensembl.org/), Genomes on a Tree (GoaT, https://goat.genomehubs.org/) and Genomes Online Database (GOLD, https://gold.jgi.doe.gov/). Our observation is that most of the eukaryotic genomes released on other platforms are eventually submitted to GenBank and become available via NCBI Datasets. However, monitoring these additional genome databases could allow for a more rapid detection of availability of genome data for particular taxon, and this could be a topic for future work.

Whole genome data is increasingly important in biological and conservation research, as it can provide a better understanding of endangered organisms, and help in the conservation efforts. Our comprehensive catalog of genomic and taxonomic information for the Japanese Red List will not only be useful for locating genome assemblies, but, importantly, it will also help focusing the future research efforts and efficiently allocating the scarce resources available for genome sequencing projects. We will continue monitoring the available data and updating our website, and similar efforts are anticipated in other regions of the world, in order to fuel preemptive, evidence-based biodiversity conservation.

## Ethics and consent

No personal or otherwise human-related data was used in this study. All data we used is already open and public. Therefore, no ethics-related or consent-related issues are applicable to this study.

## Data Availability

All our data is placed in the public domain, and shared on the website https://treethinkers.nig.ac.jp/redlist/.
