# Supplementary material for: Genome assembly catalog for species in the Japanese Red List: unlocking endangered biodiversity through genomic inventory
Source: F1000Res. 2024 Jul 22;13:583. Originally published 2024 Jun 5. [Version 2] doi: 10.12688/f1000research.149793.2 (PMC11267146; doi:10.12688/f1000research.149793.2)
Supplement: Peer review history

# Reviewer report: Reviewer response for version 2

**DOI:** 10.5256/f1000research.169110.r305503

**Published:** 2024-07-23

**Authors:** James Fleming

**Affiliations:** 1 Natural History Museum, Oslo, Norway

The revisions present a significant improvement, that address all of my comments about the work. Overall, the authors have crafted a useful new tool to inform future policy direction, and measure our progress through the sequencing of red list species. I'd welcome the further additions to the Red List monitor that they propose in future work - that sounds like some exciting future work!

Is the work clearly and accurately presented and does it cite the current literature?

Yes

If applicable, is the statistical analysis and its interpretation appropriate?

Yes

Are all the source data underlying the results available to ensure full reproducibility?

Yes

Is the study design appropriate and is the work technically sound?

Yes

Are the conclusions drawn adequately supported by the results?

Partly

Are sufficient details of methods and analysis provided to allow replication by others?

Yes

Reviewer Expertise:

Biodiversity Genomics, Ecdysozoa, Phylogenetics

I confirm that I have read this submission and believe that I have an appropriate level of expertise to confirm that it is of an acceptable scientific standard.

# Reviewer report: Reviewer response for version 2

**DOI:** 10.5256/f1000research.169110.r305502

**Published:** 2024-07-23

**Authors:** Guilherme Borba Neumann

**Affiliations:** 1 Wildlife Diseases, Leibniz Institute for Zoo and Wildlife Research (IZW) in the Forschungsverbund Berlin eV, Berlin, Berlin, Germany

Thank you the authors for addressing all my previous comments. I have no further comments and accept the paper.

Is the work clearly and accurately presented and does it cite the current literature?

Partly

If applicable, is the statistical analysis and its interpretation appropriate?

Not applicable

Are all the source data underlying the results available to ensure full reproducibility?

Yes

Is the study design appropriate and is the work technically sound?

Partly

Are the conclusions drawn adequately supported by the results?

Partly

Are sufficient details of methods and analysis provided to allow replication by others?

Yes

Reviewer Expertise:

Bioinformatics, Conservation Genetics

I confirm that I have read this submission and believe that I have an appropriate level of expertise to confirm that it is of an acceptable scientific standard.

# Reviewer report: Reviewer response for version 1

**DOI:** 10.5256/f1000research.164296.r292685

**Published:** 2024-06-25

**Authors:** Guilherme Borba Neumann

**Affiliations:** 1 Wildlife Diseases, Leibniz Institute for Zoo and Wildlife Research (IZW) in the Forschungsverbund Berlin eV, Berlin, Berlin, Germany

The current work proposes a simple way of summarizing current numbers and efforts on available whole-genome sequence information for endangered Japanese species. Although simple, the developed website provides a clear picture of needed efforts on the sequencing of most  endangered species in Japan, generating a base for further studies and ultimate impact on the conservation of endangered species. 

 Nevertheless, I suggest a few changes in the manuscript:

 In the introduction I would add some information about the total number of species in Japan, and also endemic Japanese species in order to compare with the number of species in the red list.

 It would be also interesting to know from the current available assemblies, which are annotated and presented as a Reference Genome, rather than just an assembly. Further information about the quality of the assemblies, such as number of contigs, N50 etc would also be interesting to have.

 Have the authors considered ways of automating the updates ? So that this nice work does not stop here, but guarantees a continuous monitoring of the development of genomics in endangered species.

 Other databases were left out, such as ensembl ( https://rapid.ensembl.org/index.html), which now has a rapid version of publishing new assemblies, and GOAT ( https://goat.genomehubs.org/). Actually the work here described is very similar to what has been done on GOAT. Therefore, I would suggest adding some discussion on similar efforts, maybe even comparing how well or bad represented are the Japanese species in those projects.

 Finally, I would rather add the actual figures in the manuscript than screenshots. The quality of screenshots are inferior, and it does not look very professional. In addition, it is important to add the meaning of the abbreviations in the figures as part of the figure description/legends as well.

Is the work clearly and accurately presented and does it cite the current literature?

Partly

If applicable, is the statistical analysis and its interpretation appropriate?

Not applicable

Are all the source data underlying the results available to ensure full reproducibility?

Yes

Is the study design appropriate and is the work technically sound?

Partly

Are the conclusions drawn adequately supported by the results?

Partly

Are sufficient details of methods and analysis provided to allow replication by others?

Yes

Reviewer Expertise:

Bioinformatics, Conservation Genetics

I confirm that I have read this submission and believe that I have an appropriate level of expertise to confirm that it is of an acceptable scientific standard, however I have significant reservations, as outlined above.

# Response

**Published:** 2024-07-12

**Authors:** Kirill Kryukov

**Affiliations:** Bioinformation and DDBJ Center, National Institute of Genetics, Mishima, Shizuoka, Japan

We sincerely appreciate the kind comments and suggestions. We have modified the website and revised the manuscript. Point-by-point replies:

***Reviewer Comment:***

*1. In the introduction I would add some information about the total number of species in Japan, and also endemic Japanese species in order to compare with the number of species in the red list.*

**Author Response:**

  - Thank you for the suggestion. We added the number of taxa evaluated for the Red Lists, in the Introduction. As for the total number of species in Japan, there exists an estimate of about 90,000 species, but it is based on literature in the 1990s and is not up-to-date, so we decided to not include this less reliable number.

***Reviewer Comment:***

*2. It would be also interesting to know from the current available assemblies, which are annotated and presented as a Reference Genome, rather than just an assembly. Further information about the quality of the assemblies, such as number of contigs, N50 etc would also be interesting to have.*

**Author Response:**

  - Thank you for the suggestion. We added this data to the tables. We also note that other information about each genome assembly can be found easily, since the data in our tables also serves as a link to the corresponding genome in the NCBI Datasets.

***​​​​​​​Reviewer Comment:***

*3. Have the authors considered ways of automating the updates ? So that this nice work does not stop here, but guarantees a continuous monitoring of the development of genomics in endangered species.*

**Author Response:**

  - Thank you for the suggestion. Indeed, we already automated the updates, and we are performing them periodically, usually about once a month.

***​​​​​​​Reviewer Comment:***

*4. Other databases were left out, such as ensembl ( https://rapid.ensembl.org/index.html), which now has a rapid version of publishing new assemblies, and GOAT ( https://goat.genomehubs.org/). Actually the work here described is very similar to what has been done on GOAT. Therefore, I would suggest adding some discussion on similar efforts, maybe even comparing how well or bad represented are the Japanese species in those projects.*

**Author Response:**

  - Thank you for the comment. We agree that it could be useful to monitor other genome databases. We focus on GenBank genomes as it is the largest repository of genome data, and genomes released elsewhere tend to get submitted to GenBank eventually. We added a comment about this in a discussion, and we suggest that this could be a topic of improvement in the future.

***​​​​​​​Reviewer Comment:***

*5. Finally, I would rather add the actual figures in the manuscript than screenshots. The quality of screenshots are inferior, and it does not look very professional. In addition, it is important to add the meaning of the abbreviations in the figures as part of the figure description/legends as well.*

 ​​​​​​​ **Author Response:**

  - Thank you for the suggestion. We removed the distracting borders from the figures. Our data is presented via a website, which readers will see with a web browser, therefore screenshots are the best representation of what readers will find when they explore our resource. We added the meaning of all abbreviations to figure legends.

# Reviewer report: Reviewer response for version 1

**DOI:** 10.5256/f1000research.164296.r288051

**Published:** 2024-06-20

**Authors:** James Fleming

**Affiliations:** 1 Natural History Museum, Oslo, Norway

Major Comments:

 The authors present a new resource for the scientific community - a website that tracks the progress of the sequencing of Japanese Red List species. In addition, they analyse the distribution of sequence availability for these species at the time of the resource's foundation. Overall, I believe that https://treethinkers.nig.ac.jp/redlist/ is a fascinating, worthwhile and useful tool for the scientific community, and appreciate efforts to link biodiversity genomics to conservation biology, particularly considering the precarious nature of many ecosystems in the present day. The manuscript itself is well-written, and the analysis of existing Japanese Red List data is thorough and eye-opening. I also appreciate the authors acknowledging the wide variety of scripts employed throughout the project, especially in the design and presentation of the figures.

 However, I do think that there are two important areas which go unaddressed in the manuscript in its current form.

 1) The focus on the Japanese Red List is useful for defining the scope of the project, but Japan maintains two Red Lists concurrently: one for terrestrial and freshwater organisms, and one for marine. Although it would be an extension of the scope of the manuscript as presented, expanding the tool to include the Marine Red List would be a huge boon to the proposal, especially considering the size of Japan's coastline, and the diversity of life inside it.

 2) More conceptually, I have reservations about the use of the Red List to direct genome sequencing efforts in the near future, as the authors propose. The manuscript feels like it is missing a section in both its introduction and discussion where it considers the flaws of Red List methodologies, and the limitations that they might present to the tool. The Red List is very heavily biased towards larger organisms, and particularly vertebrates, meaning that the existing mammal and bird bias already noted by the authors in the results section of the manuscript is in actuality a bias within a bias. My concern is that directing policy towards "completing the Red List" might actually result in more species falling through the cracks, particularly in areas of biology that are already chronically underfunded. There is a strong body of literature on this subject, and it would greatly improve the manuscript to address it - I've added some useful references below that might help as a starting point:

 Possingham H.et.al.,2002 (ref 1)

 Cazalis V et.al., 2022 (Ref 2)

 Régnier C, et.al., 2009 (ref 3)

 Goodsell R et.al., 2024 (ref 4)

 That said, I do believe that this tool is a potentially incredibly useful resource for researchers of Red List species, and I appreciate efforts to unite the two disciplines towards their closely connected goals.

 Very Minor Comments:

 Abstract:

 "are allowing the dramatic increase of whole genome data for a wide variety of species." -> "are allowing for the dramatic increase of whole genome data for a wide variety of species."

 "genome sequence data can assist the monitoring of intraspecific genetic diversity" -> "genome sequence data can assist in the monitoring of intraspecific genetic diversity"

 "The combined data shows a low percentage (2.1%) of the availability of whole genome sequence data for the taxa ranked on the Japanese Red List" -> "The combined data shows that whole genome sequence data is available for a low percentage (2.1%) of the taxa ranked on the Japanese Red List"

 "instructs future policies including which taxon needs more effort for genome sequencing." -> "instructs future policies, including which taxa require greater effort in genome sequencing."

 Introduction:

 "Genome DNA sequences do not only instruct cellular and other phenomena through genetic readout but also contain the valuable information of genetic diversity when they are analyzed on a population level." -> " but also contain valuable information about genetic diversity when they are analyzed on a population level."

 "some projects under the EBP are dedicated to promote this trend" -> " some projects under the EBP are dedicated to promoting this trend"

 Discussion:

 " It is crucial that 8.9% of Red List entries are not registered in any of the three taxonomic databases that we used." - I wasn't quite sure what this sentence was trying to be highlight, so it might be useful to rephrase it.

 " the taxon Amphibia in our list contains quite a few salamanders" -> how many? I think a number could really help support the overall thrust of the point.

 " The technical difficulty is reflected in the unavailability of genome assemblies in this taxon." -> " The technical difficulty is reflected in the lack of available genome assemblies for this taxon."

Is the work clearly and accurately presented and does it cite the current literature?

Yes

If applicable, is the statistical analysis and its interpretation appropriate?

Yes

Are all the source data underlying the results available to ensure full reproducibility?

Yes

Is the study design appropriate and is the work technically sound?

Yes

Are the conclusions drawn adequately supported by the results?

Partly

Are sufficient details of methods and analysis provided to allow replication by others?

Yes

Reviewer Expertise:

Biodiversity Genomics, Ecdysozoa, Phylogenetics

I confirm that I have read this submission and believe that I have an appropriate level of expertise to confirm that it is of an acceptable scientific standard, however I have significant reservations, as outlined above.

## References

[1] : Limits to the use of threatened species lists. *Trends in Ecology & Evolution* .2002;17(11) : 10.1016/S0169-5347(02)02614-9 503-507 10.1016/S0169-5347(02)02614-9

[2] : Bridging the research-implementation gap in IUCN Red List assessments. *Trends Ecol Evol* .2022;37(4) : 10.1016/j.tree.2021.12.002 359-370 10.1016/j.tree.2021.12.002 35065822

[3] : Not knowing, not recording, not listing: numerous unnoticed mollusk extinctions. *Conserv Biol* .2009;23(5) : 10.1111/j.1523-1739.2009.01245.x 1214-21 10.1111/j.1523-1739.2009.01245.x 19459894

[4] : The rarity of Invertebrates prevents reliable application of IUCN Red-List criteria. 2024; 10.32942/X23G71 10.32942/X23G71

# Response

**Published:** 2024-07-12

**Authors:** Kirill Kryukov

**Affiliations:** Bioinformation and DDBJ Center, National Institute of Genetics, Mishima, Shizuoka, Japan

We sincerely appreciate the encouraging comments and discussion. We have modified the website and revised the manuscript. Point-by-point replies:

**Reviewer Comment:**

*1) The focus on the Japanese Red List is useful for defining the scope of the project, but Japan maintains two Red Lists concurrently: one for terrestrial and freshwater organisms, and one for marine. Although it would be an extension of the scope of the manuscript as presented, expanding the tool to include the Marine Red List would be a huge boon to the proposal, especially considering the size of Japan's coastline, and the diversity of life inside it.*

***Author Response:***

  - Thank you for the nice suggestion. We now incorporated the Marine Red List data to our analysis, and we agree that the resulting resource is more complete and interesting.

**Reviewer Comment:**

*2) More conceptually, I have reservations about the use of the Red List to direct genome sequencing efforts in the near future, as the authors propose. The manuscript feels like it is missing a section in both its introduction and discussion where it considers the flaws of Red List methodologies, and the limitations that they might present to the tool. The Red List is very heavily biased towards larger organisms, and particularly vertebrates, meaning that the existing mammal and bird bias already noted by the authors in the results section of the manuscript is in actuality a bias within a bias. My concern is that directing policy towards "completing the Red List" might actually result in more species falling through the cracks, particularly in areas of biology that are already chronically underfunded. There is a strong body of literature on this subject, and it would greatly improve the manuscript to address it - I've added some useful references below that might help as a starting point:*

*Possingham H.et.al.,2002 (ref 1)*

*Cazalis V et.al., 2022 (Ref 2)*

*Régnier C, et.al., 2009 (ref 3)*

*Goodsell R et.al., 2024 (ref 4)*

***Author Response:***

  - Thank you very much for your suggestion. We have modified the relevant parts to draw readers' attention to criticisms of biased species selection.

**Reviewer Comment:**

*Very Minor Comments: .......genome assemblies for this taxon."*

***Author Response to Reviewer's Minor Comments:***

  - We appreciate all minor comments, and we addressed them in the revised manuscript.
